# Anatomic Reconstruction of Acute Posterior Shoulder Instability With Reverse Hill-Sachs Lesions: A Report of Two Cases

**DOI:** 10.7759/cureus.116191

**Published:** 2026-09-13

**Authors:** Jorge Vélez, Corina E Salas, Jorge Rojas, Guido A Fierro, Juan C Gonzalez

**Affiliations:** 1 Department of Orthopedics and Traumatology - Shoulder and Elbow Surgery, Fundación Santa Fe de Bogotá, Bogotá, COL; 2 Department of Orthopedics and Traumatology, Universidad del Rosario, Bogotá, COL; 3 School of Medicine, Universidad de los Andes, Bogotá, COL

**Keywords:** arthroscopic-open approach, humeral head defect, joint-preserving surgery, posterior shoulder instability, reverse hill-sachs lesion

## Abstract

Posterior shoulder dislocation associated with a reverse Hill-Sachs lesion is an uncommon injury that may result in persistent instability when the humeral head defect is engaged. Management depends on lesion size, engagement, chronicity, and reconstructibility; both non-anatomic procedures and anatomic reconstruction strategies have been described for acute lesions within the joint-preserving range. We present two cases of acute posterior shoulder instability with engaging reverse Hill-Sachs lesions treated with a combined arthroscopic-open approach. In both patients, arthroscopic posterior labral repair was followed by open disimpaction and graft-supported anatomic reconstruction of the humeral head through a deltopectoral approach with partial superior subscapularis tenotomy. In the first case, supplemental cannulated screw fixation (Raynham, MA: DePuy Synthes) was used because of a deeper defect, whereas in the second case graft support alone was sufficient after stable reduction was achieved. Immediate post-operative imaging demonstrated concentric reduction of the glenohumeral joint and restoration of humeral head contour in both patients. At a final follow-up of 24 months, neither patient had sustained recurrent instability or other symptoms, and neither required reoperation or other intervention. Active forward elevation was 160° in both patients, with external rotation at the side of 90° and 70° and internal rotation to T7 and T9, respectively. American Shoulder and Elbow Surgeons scores were 90 and 88, Single Assessment Numeric Evaluation scores 95 and 93, and visual analog scale pain scores 0 of 10 in both patients. This report highlights a practical combined arthroscopic-open, joint-preserving strategy that addresses both the posterior capsulolabral lesion and the humeral head defect while allowing direct visualization of the impacted articular surface and fixation tailored to lesion morphology. In selected acute, engaging, and reconstructible reverse Hill-Sachs lesions, this approach may be technically feasible as a joint-preserving anatomic option; comparative studies are required before it can be positioned as an alternative to established non-anatomic procedures.

## Introduction

Posterior shoulder dislocation is an uncommon injury, but it is frequently associated with an anteromedial humeral head impaction fracture, commonly referred to as a reverse Hill-Sachs lesion [1]. When the defect is sufficiently large or engaging, it may compromise glenohumeral congruity and predispose to persistent instability or recurrent posterior subluxation after reduction. Lesion size, location, and engagement are therefore central to treatment planning, and the gamma angle has been proposed as a useful adjunct to estimate the risk of engagement in posterior instability [2,3].

Current treatment strategies are largely guided by defect magnitude, chronicity, and reconstructibility [4,5]. Small, non-engaging defects may be treated non-operatively, whereas defects exceeding approximately 45-50% of the humeral head generally require arthroplasty-based solutions [4,5]. Between these extremes lies a joint-preserving zone, typically involving acute lesions of roughly 20% to 45% or 50%, in which both non-anatomic procedures, such as the McLaughlin or modified McLaughlin techniques, and anatomic reconstruction strategies have been reported with favorable outcomes [6-10]. Recent reviews suggest that surgical treatment of reverse Hill-Sachs lesions in the setting of posterior instability is associated with low recurrence and satisfactory functional outcomes across different techniques, although the evidence remains limited to predominantly small retrospective series [1,4]. Notably, both lesions in the present report lie at the lower boundary of this joint-preserving range, at a defect magnitude for which non-operative management or isolated soft-tissue stabilization might be considered on size-based criteria alone. In both patients, the decision to proceed with humeral head reconstruction was therefore driven by demonstrated engagement and by a gamma angle above the reported threshold for engagement, rather than by defect size in isolation.

McLaughlin-type procedures remain reliable and widely used options for engaging lesions in this reconstructive range, but they are intrinsically non-anatomic because they fill the defect with transferred tendon or bone-tendon tissue rather than restoring the native humeral head contour [6,9]. By contrast, anatomic reconstruction through disimpaction and graft support aims to restore humeral head sphericity and preserve joint congruity. Previous reports have described open reconstruction with grafting and fixation, arthroscopic or arthroscopy-assisted retrograde disimpaction, and combined procedures addressing both the posterior capsulolabral lesion and the humeral head defect [7,10-14]. More recently, open anatomic reduction and fixation through a deltopectoral approach with lesser tuberosity osteotomy has been described as a joint-preserving option for acute reverse Hill-Sachs lesions, further supporting the relevance of anatomic restoration in selected cases [14].

In selected acute, engaging, and reconstructible reverse Hill-Sachs lesions, particularly in young patients, a joint-preserving anatomic strategy may offer the advantage of restoring the articular surface while simultaneously addressing associated posterior soft-tissue instability. The purpose of this report of two cases is to describe a combined technique consisting of arthroscopic posterior labral repair followed by open disimpaction and graft-supported anatomic reconstruction of the humeral head through a deltopectoral approach with partial superior subscapularis tenotomy. In contrast to the recently described open technique using lesser tuberosity osteotomy, our approach was intended to provide direct visualization and controlled reduction of the impacted articular surface while avoiding the need for lesser tuberosity osteotomy and allowing fixation to be tailored according to defect morphology and post-reduction stability.

## Case presentation

We present two cases of acute posterior shoulder instability associated with engaging reverse Hill-Sachs lesions treated with a combined strategy of arthroscopic posterior capsulolabral repair and open anatomic reconstruction of the humeral head. In both patients, the goal was to address the posterior soft-tissue lesion while restoring humeral head sphericity through open disimpaction and graft-supported reduction of the reverse Hill-Sachs defect.

Case 1

A 20-year-old man with a history of epilepsy presented with pain and limited range of motion of the right shoulder after a seizure episode. Initial evaluation demonstrated an acute posterior dislocation of the right shoulder associated with a reverse Hill-Sachs lesion. Closed reduction was performed in the emergency department; however, the shoulder spontaneously dislocated thereafter.

Plain radiographs and computed tomography (CT) confirmed posterior glenohumeral dislocation with an anteromedial humeral head impaction fracture consistent with a reverse Hill-Sachs lesion (Figures 1A-1D). The gamma angle is a standardized measurement that combines the size and the localization of a reverse Hill-Sachs lesion and describes the position of its posterior defect margin relative to the humeral head; values above the reported threshold for engagement have been associated with contact of the defect against the posterior glenoid rim and with recurrent instability after closed reduction [2,3]. In this patient, the gamma angle was measured on the axial CT image at the level of the greatest extent of the defect and was 91° (Figure 1E). The gamma angle was used prospectively in operative decision-making as follows: a value above this threshold, combined with engagement demonstrated on examination under anesthesia, was the principal indication for humeral head reconstruction despite a defect size at the lower end of the reconstructive range. Defect size was quantified on the same axial CT images using the best-fit circle method on a dedicated imaging workstation (XERO Universal Viewer; Mortsel, Belgium: AGFA HealthCare), in which a circle was fitted to the preserved articular contour of the humeral head, and the defect was expressed as the proportion of the articular arc lost. All measurements were performed by a senior author. The estimated humeral head defect involved approximately 20% of the articular surface. Associated posterior glenoid bone loss was not identified. The patient was temporarily immobilized in external rotation to maintain reduction.

**Figure 1:**
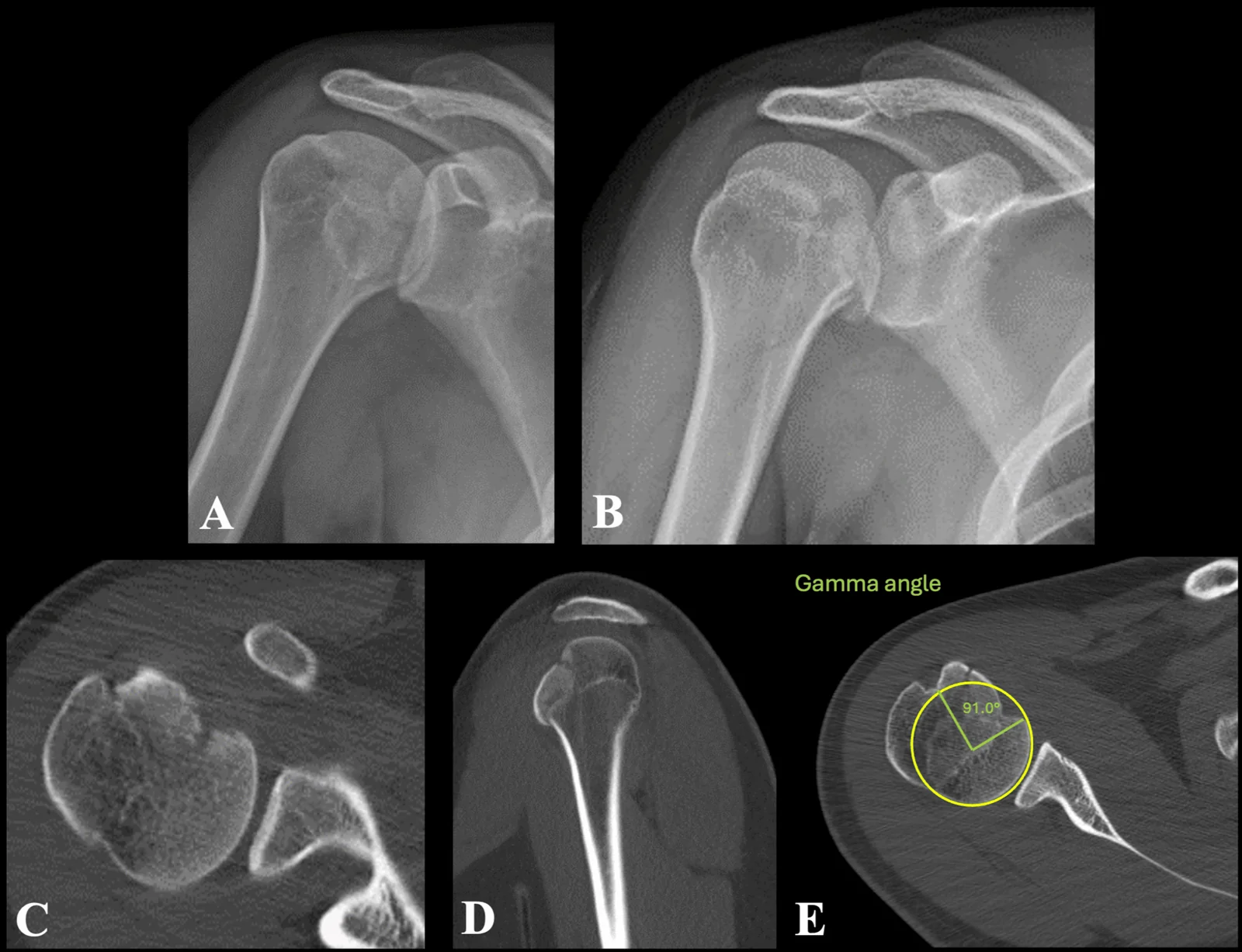
Pre-operative imaging of Case 1. (A, B) Plain radiographs demonstrating posterior dislocation of the right shoulder. (C, D) Axial and sagittal CT images demonstrating an anteromedial humeral head impaction fracture consistent with a reverse Hill-Sachs lesion. (E) Gamma angle measurement of 91°.

Given the patient’s young age, acute presentation, recurrent instability after closed reduction, engaging lesion, and associated posterior instability pattern, surgical treatment was indicated. Time from injury to surgery was 14 days.

Under general anesthesia and after an ultrasound-guided interscalene block, the patient was positioned in the beach-chair position. Examination under anesthesia demonstrated marked posterior translation of the humeral head with engagement of the reverse Hill-Sachs defect against the posterior glenoid, reproducing articular locking. Arthroscopy was performed first using one anterosuperior viewing portal and two working portals (anterior and posterior). A posteroinferior labral lesion was identified and repaired using three 2.9 mm knotless anchors, each loaded with 1.5 mm suture tape, passed through the posterior labrum and capsule. Dynamic arthroscopic examination after completion of the repair confirmed restoration of posterior capsulolabral stability. However, with the arm in adduction and internal rotation and a posteriorly directed load applied, the reverse Hill-Sachs defect continued to engage against the posterior glenoid rim, reproducing articular locking. Persistent engagement in this position after completion of the labral repair was the intra-operative criterion used to proceed with humeral head reconstruction; had the defect not engaged throughout the functional arc, the procedure would have been concluded after the arthroscopic repair.

Attention was then turned to the humeral head defect. Through a deltopectoral approach, a partial tenotomy of the superior 1 cm of the subscapularis tendon was performed to expose the anteromedial impaction fracture. The reverse Hill-Sachs defect was identified under direct visualization as an impacted anteromedial osteochondral lesion (Figure 2A). With the arm internally rotated, an anterior cruciate ligament reconstruction C-guide was centered over the defect and aligned with the posterior portal trajectory at approximately 90°. An 8 mm drill was used to create a tunnel from the posterolateral humeral cortex toward the defect. The impacted osteochondral segment was then elevated under direct visualization using 8 mm dilators until anatomic reduction of the articular contour was achieved (Figure 2B). The subchondral void was filled with morselized corticocancellous allograft obtained from our institutional bone bank (Fundación Cosme y Damián, Banco de Huesos y Tejidos, Bogotá, Colombia) and supplied in 20 cc vials; a single vial was sufficient in each case. The graft was delivered through the retrograde tunnel and impacted in successive increments with a bone tamp until the elevated osteochondral segment was fully supported. Adequate subchondral support and graft containment were confirmed intra-operatively by direct visualization of a restored and congruent articular contour without residual step-off and by the absence of graft extrusion at the articular margin. Because the defect was deeper and required additional support, two 4.5-mm partially threaded cannulated screws (Raynham, MA: DePuy Synthes) were placed from an anterolateral approach as subchondral support screws beneath the reconstructed articular surface. The threaded portion of each screw engaged the subchondral bone supporting the elevated osteochondral segment, with the screw heads seated on the lateral cortex and without articular penetration. At the end of the procedure, the partial superior subscapularis tenotomy was repaired tendon-to-tendon using modified Mason-Allen stitches. Repair integrity was confirmed under direct vision with the arm brought through a passive external rotation arc before closure.

**Figure 2:**
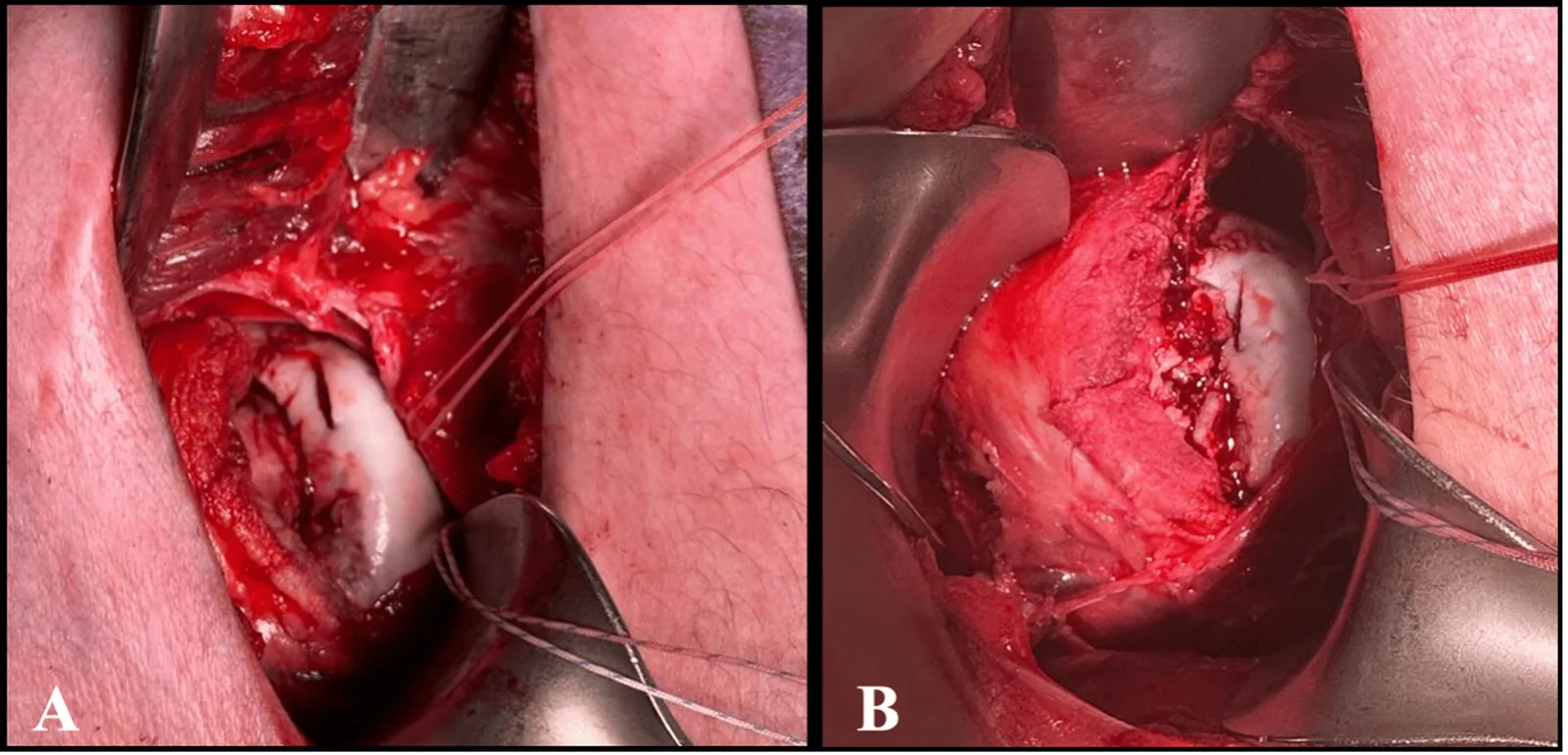
Intra-operative images of Case 1. (A) Direct visualization of the impacted anteromedial reverse Hill-Sachs lesion after deltopectoral exposure and partial superior subscapularis tenotomy. (B) Appearance after disimpaction and restoration of the articular contour.

Immediate post-operative anteroposterior and axillary radiographs confirmed concentric reduction of the glenohumeral joint, restoration of the humeral head contour, and satisfactory placement of the screws (Figures 3A, 3B). The patient was discharged on post-operative day one with the arm immobilized in neutral rotation.

**Figure 3:**
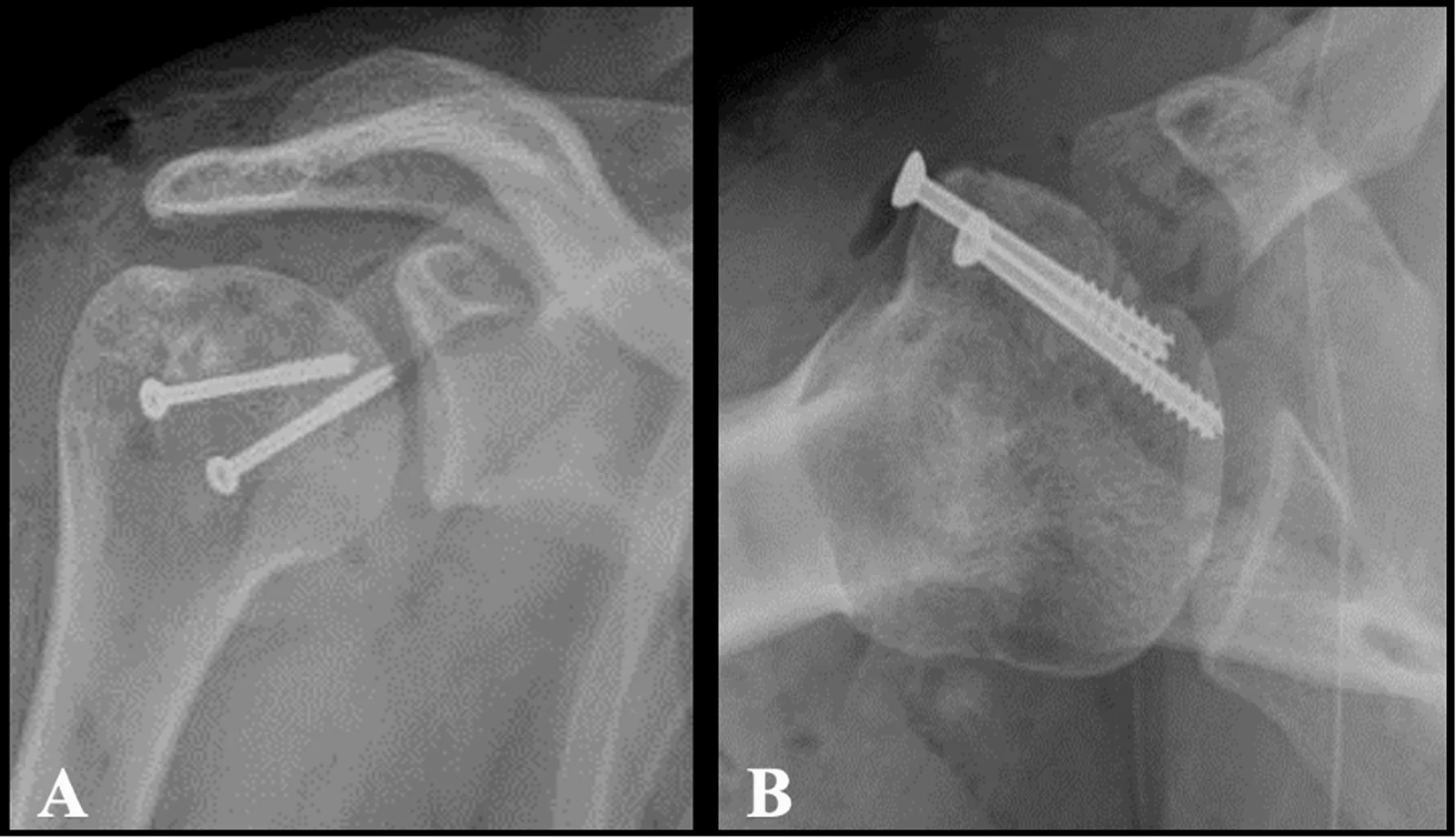
Immediate post-operative radiographs of Case 1. (A) Anteroposterior view demonstrating concentric reduction of the glenohumeral joint and satisfactory placement of cannulated screws from an anterolateral direction. (B) Axillary view demonstrating concentric reduction and adequate placement and alignment of screws.

At the final follow-up of 24 months, the patient reported no pain and had no episodes of recurrent instability. Active forward elevation was 160°, external rotation at the side was 90°, and internal rotation reached T7. Functional outcome scores were as follows: American Shoulder and Elbow Surgeons (ASES) score 90, Single Assessment Numeric Evaluation (SANE) score 95, and visual analog scale (VAS) pain score 0/10. Follow-up radiographs demonstrated maintenance of reduction with graft incorporation and no evidence of collapse or hardware complications. The cannulated screws remained in situ at final follow-up, with no radiographic evidence of loosening, articular penetration, or symptomatic prominence. Routine hardware removal was not planned and has not been performed; the screws were retained because they were fully contained within the subchondral bone and remained asymptomatic. Removal would be considered only if the patient developed hardware-related symptoms.

Case 2

A 22-year-old man sustained high-energy trauma in a motor vehicle accident, resulting in a diaphyseal femoral fracture and an ipsilateral posterior dislocation of the right shoulder. The femoral fracture was treated with intramedullary nailing by the orthopedic trauma team. During the same hospitalization period, the shoulder injury was identified and managed with closed reduction followed by immobilization in external rotation.

Plain radiographs and magnetic resonance imaging demonstrated a reverse Hill-Sachs lesion involving the anteromedial humeral head, associated with a posterior labral tear (Figures 4A, 4B). The gamma angle measured 93° (Figure 4C). The estimated defect size involved approximately 18% of the humeral head articular surface. Associated posterior glenoid bone loss was not identified. Time from injury to surgery was 17 days. A dedicated CT of the shoulder was not obtained in this patient. The shoulder injury was identified during the initial management of a high-energy polytrauma, and magnetic resonance imaging was performed to characterize the suspected posterior labral injury; because this study also demonstrated the impaction defect adequately, and because the standardized measurements used here have been reported on both computed tomography and magnetic resonance images, no additional cross-sectional imaging was requested [15,16]. Both the gamma angle and the defect size in this patient were therefore measured on the axial magnetic resonance images at the level of greatest defect extent, using the same method applied to the CT images in Case 1. The limitation arising from the use of different imaging modalities between the two cases is addressed in the following section.

**Figure 4:**
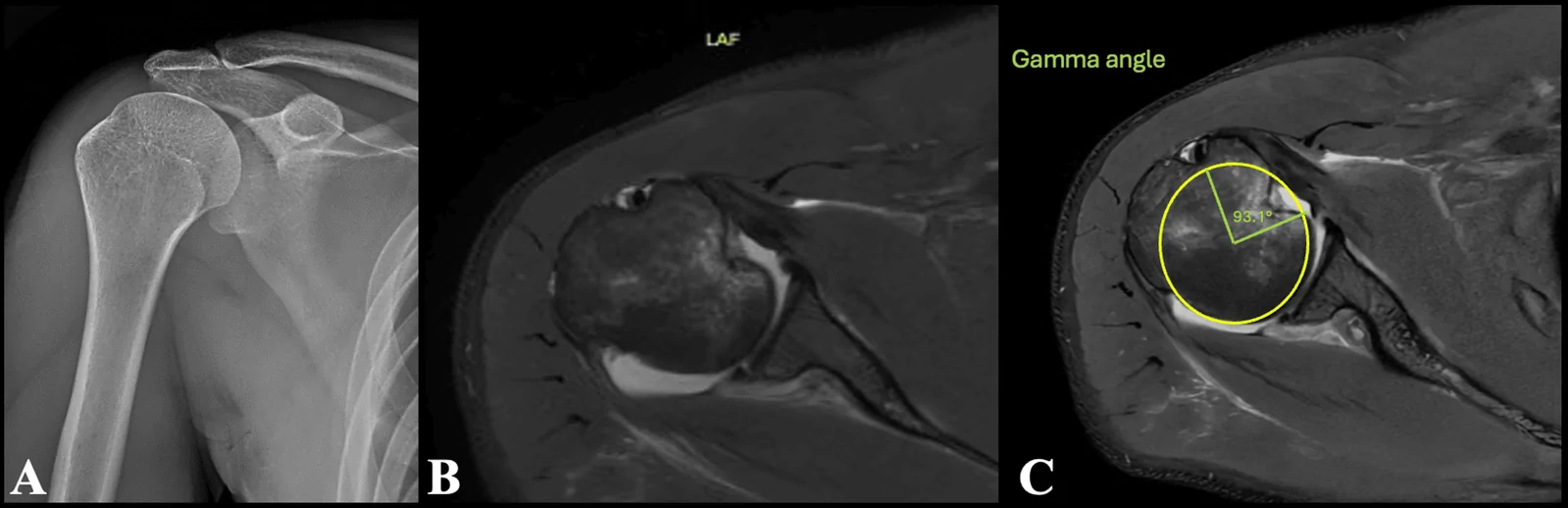
Pre-operative imaging of Case 2. (A) Anteroposterior radiograph demonstrating posterior shoulder dislocation with an associated reverse Hill-Sachs lesion. (B) Axial magnetic resonance image demonstrating the reverse Hill-Sachs lesion and associated posterior labral tear. (C) Gamma angle measurement of 93°.

Given the patient’s young age, acute lesion, posterior labral injury, and engaging reverse Hill-Sachs defect, operative treatment was recommended in order to restore stability and preserve humeral head anatomy. Surgery was performed in the beach-chair position under general anesthesia with an ultrasound-guided interscalene block. Examination under anesthesia confirmed posterior translation and engagement of the humeral head, which was manually reducible. Arthroscopic evaluation was then carried out using the same three-portal configuration as in Case 1. A posteroinferior labral lesion was identified and repaired with three 2.9 mm knotless anchors loaded with 1.5 mm suture tape. Dynamic arthroscopic assessment demonstrated restoration of posterior capsulolabral stability.

A deltopectoral approach was then used to address the reverse Hill-Sachs lesion. After partial tenotomy of the superior 1 cm of the subscapularis tendon, the anteromedial defect was exposed under direct visualization. A retrograde tunnel was created from the posterolateral humeral cortex toward the defect using an anterior cruciate ligament (ACL) C-guide and an 8 mm drill. The impacted segment was elevated with 8-mm dilators until restoration of the humeral head contour was achieved, and the resulting subchondral void was filled with the same corticocancellous allograft, prepared, delivered, and impacted as described for Case 1, with the same intra-operative confirmation of subchondral support and graft containment. Compared with Case 1, this lesion was less deep, and after disimpaction and graft support, the reduction was considered stable; therefore, no supplemental screw fixation was used. Immediate post-operative anteroposterior and axillary radiographs demonstrated satisfactory reduction of the dislocation and restoration of humeral head sphericity (Figures 5A, 5B). The shoulder was immobilized in neutral rotation.

**Figure 5:**
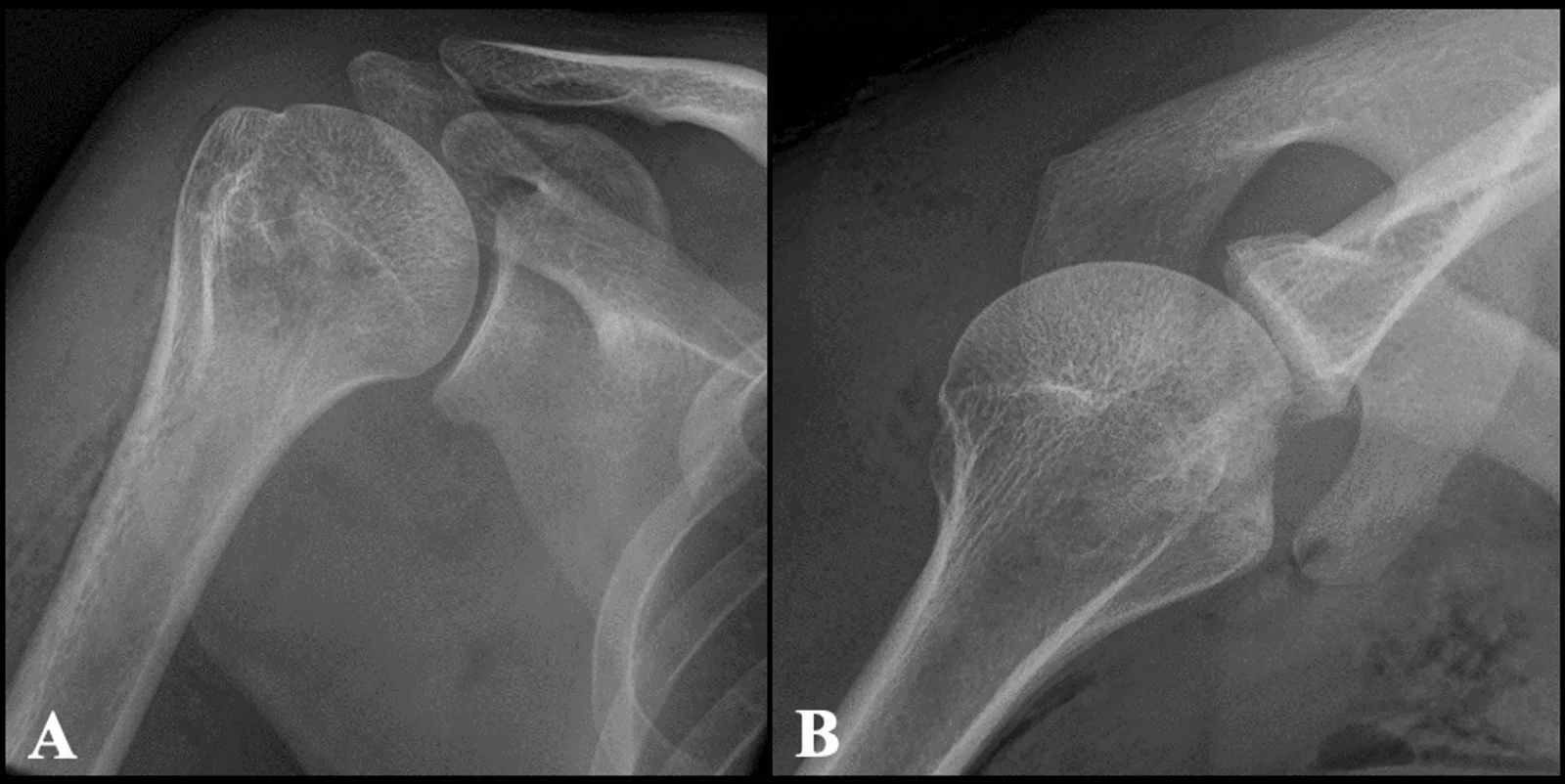
Immediate post-operative shoulder radiographs. (A) Anteroposterior view demonstrating concentric reduction of the glenohumeral joint. (B) Axillary view demonstrating satisfactory reduction of the glenohumeral joint and restoration of the humeral head contour.

At the final follow-up of 24 months, the patient reported no pain and had no episodes of recurrent instability. Active forward elevation was 160°, external rotation at the side was 70°, and internal rotation reached T9. Functional outcome scores were as follows: ASES score 88, SANE score 93, and VAS pain score 0/10. Follow-up radiographs demonstrated maintenance of reduction with graft incorporation and no evidence of collapse or hardware.

Post-operative management

The post-operative protocol was identical in both patients. It was designed to protect the posterior capsulolabral repair and the partial superior subscapularis repair simultaneously; because these two repairs are stressed by opposite motions, the corresponding restrictions were applied together rather than in isolation. The shoulder was immobilized in a sling in neutral or slight external rotation for four weeks. Passive range of motion was begun at two weeks, active-assisted motion at four weeks, unrestricted active motion at six weeks, and strengthening at 12 weeks. During the first six weeks, forward elevation was limited to 90° until week four and to 120° until week six; external rotation at the side was limited to 30° to protect the subscapularis repair; and internal rotation beyond the abdomen and cross-body adduction were avoided entirely to protect the posterior capsulolabral repair. The combined position of flexion, adduction, and internal rotation, in which the defect engaged intra-operatively, was specifically prohibited until 12 weeks. Resisted internal rotation was not permitted before 12 weeks. Activities of daily living were allowed from two weeks and were limited to the use of the hand for eating, writing, and personal hygiene, with the elbow held at the side and the sling in place, without lifting, reaching away from the body, or weight-bearing through the operated limb. Return to work was permitted at 12 weeks and return to sport or higher-demand activity at six months. The protocol is summarized by post-operative phase in Table 1. No post-operative complications such as infection, neurologic deficits, wound problems, recurrent instability, stiffness requiring intervention, hardware failure, or reoperation were observed.

**Table 1:** Post-operative rehabilitation protocol by phase.

Post-operative phase	Immobilization	Permitted motion	Restrictions protecting the posterior capsulolabral repair	Restrictions protecting the partial subscapularis repair	Strengthening/functional milestone
0-2 weeks	Sling in neutral or slight external rotation, worn continuously except for hygiene	Elbow, wrist, and hand motion; pendulum exercises only	No internal rotation; no cross-body adduction; no forward flexion beyond 60°	No active internal rotation; passive external rotation limited to 30°	None
2-4 weeks	Sling continued	Passive forward elevation to 90°; passive external rotation to 30°	No internal rotation beyond neutral; no cross-body adduction	Passive external rotation not beyond 30°; no resisted internal rotation	Activities of daily living with the elbow at the side (text)
4-6 weeks	Sling discontinued at 4 weeks	Active-assisted forward elevation to 120°; external rotation to 40°	Internal rotation to the abdomen only; no cross-body adduction	No resisted internal rotation; external rotation progressed gradually	Scapular setting and isometric exercises
6-12 weeks	None	Progression to full active range of motion in all planes	Avoid end-range flexion-adduction-internal rotation loading until 12 weeks	Gentle active internal rotation permitted; no resisted internal rotation until 12 weeks	Rotator cuff and periscapular endurance work without resistance in internal rotation
12 weeks-6 months	None	Full range of motion	None	Progressive resisted internal rotation from 12 weeks	Progressive resistance training; return to work at 12 weeks
At 6 months	None	Full range of motion	None	None	Return to sport or higher-demand activity

## Discussion

Posterior shoulder dislocation associated with an engaging reverse Hill-Sachs lesion remains a challenging injury because treatment must address both glenohumeral stability and restoration of the humeral articular surface [1,3]. Management is primarily guided by lesion size, chronicity, engagement, and reconstructibility. Small, non-engaging defects may be treated non-operatively in selected cases, whereas larger lesions, particularly those involving approximately 45-50% or more of the humeral head, may require arthroplasty-based solutions [4,5]. Between these extremes lies a joint-preserving zone in which acute engaging lesions of intermediate size may be treated with either non-anatomic procedures, such as the McLaughlin or modified McLaughlin techniques, or with anatomic reconstruction strategies [4,5,17].

McLaughlin-type procedures remain reliable and widely used options for engaging reverse Hill-Sachs lesions and have shown good clinical outcomes with low recurrence in published series [6,8,9]. However, these techniques are intrinsically non-anatomic because they fill the defect with transferred soft tissue or bone-tendon tissue rather than restoring the native humeral head contour. In young patients with acute, reconstructible lesions, anatomic reconstruction may therefore offer a theoretical advantage by preserving humeral head sphericity and joint congruity while avoiding alteration of the subscapularis footprint or articular geometry [4,10]. In the two patients presented, a McLaughlin or modified McLaughlin procedure was considered but not selected. Both lesions were acute and of small to moderate size and consisted of a well-defined impacted osteochondral segment that appeared amenable to elevation, so that the native articular surface could be restored directly rather than filled with transferred tissue. Both patients were also young, and transfer of the subscapularis tendon or of the lesser tuberosity alters the subscapularis footprint and may limit internal rotation, which we preferred to avoid in the absence of an irreparable defect or of non-viable articular cartilage. Finally, both patients had a posterior capsulolabral lesion that required arthroscopic repair, and the combined strategy allowed the soft-tissue and osseous components of the injury to be addressed without sacrificing the anterior structures.

Previous reports of anatomic reconstruction for reverse Hill-Sachs lesions have described different technical strategies. Open reconstruction with reduction, graft support, and fixation has been reported by Banerjee et al. and Bock et al., whereas arthroscopic or arthroscopy-assisted disimpaction has been described by Bark et al. and more recently by Festbaum et al. [7,11-13]. Recent literature has further supported open anatomical reduction and fixation through a deltopectoral approach as a joint-preserving option in acute cases, including a technique that uses lesser tuberosity osteotomy for exposure and fixation of the reduced osteochondral fragment [14]. Taken together, these reports suggest that anatomic restoration is feasible in selected acute lesions, but the available evidence remains limited to small retrospective series and technical reports rather than comparative studies [4,14].

The main contribution of the present report is not the introduction of an entirely new reconstructive concept, but rather the description of a practical combined arthroscopic-open strategy for acute posterior instability involving engagement of reverse Hill-Sachs lesions. In both patients, we addressed the posterior capsulolabral lesion arthroscopically and then performed open disimpaction and graft-supported reconstruction of the humeral head. We believe this combined approach is important because these injuries are frequently bipolar, with both a posterior soft-tissue lesion and an engaging humeral head defect contributing to instability [18]. Addressing only one component may leave residual instability or persistent risk of engagement.

A specific technical feature of our approach is the use of a deltopectoral exposure with partial superior subscapularis tenotomy to directly visualize the anteromedial defect. Arthroscopic disimpaction has the advantage of minimal invasiveness and has shown encouraging results in selected acute lesions [7,12]. Nevertheless, we elected to use an open approach for the humeral head reconstruction because it provided direct visualization of the impacted articular surface, facilitated controlled elevation of the depressed osteochondral segment, and allowed straightforward placement of cancellous graft. In contrast to the recently described open technique using lesser tuberosity osteotomy, our exposure was obtained through a limited partial subscapularis tenotomy, which we considered sufficient to access the defect while avoiding the need for osteotomy and its associated fixation [14]. We consider this to be a practical technical variation rather than a claim of superiority.

Another relevant point in our series is that fixation was tailored to defect morphology. In the first case, the lesion appeared deeper after disimpaction and required additional subchondral support; therefore, two cannulated screws were placed as subchondral support to maintain the reduced articular segment. In the second case, the defect was smaller and shallower, and after disimpaction and graft support, the reconstruction remained stable without supplemental fixation. This suggests that screw fixation may not be mandatory for every reconstructible reverse Hill-Sachs lesion and can instead be individualized based on defect depth and post-reduction stability. Although previous open reconstruction reports have commonly used fixation, the optimal method and indications for supplemental hardware remain incompletely defined in the literature [10,11,14].

The presented cases also support the concept that lesion size alone should not determine management. In both patients, the estimated defects were in the lower end of the reconstructive range, approximately 20% in Case 1 and 18% in Case 2, but both had gamma angles above the reported threshold for engagement (91° and 93°, respectively) and demonstrated engagement intra-operatively, making them clinically relevant despite their moderate size [3]. This emphasizes that the engagement and instability patterns may be as important as the absolute defect size when deciding among non-operative treatment, soft-tissue stabilization alone, non-anatomic filling procedures, and anatomic humeral head reconstruction.

The present report has several limitations. First, it includes only two cases, which limits generalizability. Selection and reporting bias must also be considered - only two patients are presented, both with favorable outcomes, and these results should therefore not be interpreted as representative. These were the first two patients at our institution to undergo this technique, and no cases treated with this technique during the study period were excluded. The possibility that unfavorable results are underrepresented cannot be excluded. Second, it is not a comparative study and therefore does not allow any conclusion regarding superiority over established alternatives such as the McLaughlin or modified McLaughlin procedures. Although both patients were followed for 24 months, this remains a short observation period for a reconstructive procedure, and longer-term surveillance is needed to determine whether the reconstructed humeral head maintains its contour without collapse, osteonecrosis, degenerative change, or recurrent instability. Finally, because reverse Hill-Sachs lesions are uncommon and treatment algorithms are largely based on retrospective evidence, our report should be interpreted as illustrating technical feasibility and clinical rationale rather than as definitive evidence of comparative effectiveness [4,5]. Two further limitations relate to measurement and to implant retention. Defect size and gamma angle were quantified on CT in Case 1 but on magnetic resonance imaging in Case 2, because a dedicated CT was not obtained in the second patient; CT is generally considered more accurate than MRI for quantifying osseous defects, and this difference in modality limits the direct comparability of the measurements reported for the two cases. All imaging measurements were performed by a single observer; therefore, inter- and intra-observer reliability were not assessed. In addition, the cannulated screws used in Case 1 were retained, and the long-term consequences of permanent subchondral implant retention in a 20-year-old patient, including the possible need for later removal, cannot be assessed within the present follow-up period.

Despite these limitations, we believe that these cases add useful information to the current literature. Specifically, they show that acute engaging reverse Hill-Sachs lesions can be managed with a combined arthroscopic-open strategy that addresses both the posterior labral injury and the humeral head defect, while using a limited anterior exposure for direct visualization and tailoring fixation to lesion morphology. In selected young patients with acute, reconstructible lesions, these cases suggest the feasibility of a joint-preserving anatomic reconstruction in this setting; whether it confers any advantage over non-anatomic procedures cannot be determined from this series and warrants further comparative study [7,10,14].

## Conclusions

In selected acute, engaging, and reconstructible reverse Hill-Sachs lesions, combined arthroscopic posterior labral repair and open graft-supported disimpaction may offer a technically feasible, joint-preserving option warranting further comparative study. This report of two patients describes technical feasibility and short-term clinical results; it does not establish the role of this technique relative to McLaughlin-type or other established procedures, and comparative studies with larger samples and longer follow-up are required before any such conclusion can be drawn.
